# From Frenulum Tear to Transfusion—An Unexpected Diagnosis of Hemophilia in a Toddler: A Case Report

**DOI:** 10.5811/cpcem.63677

**Published:** 2026-10-20

**Authors:** Saxony Busta, Mary Jane Piroutek

**Affiliations:** Rady Children’s Health Orange County, Department of Pediatric Emergency Medicine, Orange, California; University of California Irvine

**Keywords:** hemophilia A, labial frenulum tear, pediatric bleeding, nonaccidental trauma differential, case report

## Abstract

**Introduction:**

Labial frenulum tears in infants are commonly associated with minor trauma but may raise concern for nonaccidental injury. These injuries are typically benign and self-limiting; however, persistent or excessive bleeding should prompt evaluation for an underlying bleeding disorder. Hemophilia A is an X-linked factor VIII deficiency with clinical symptoms that vary based on the level of severity.

**Case Report:**

A previously healthy 14-month-old male presented to the pediatric emergency department three times within 10 days for persistent bleeding from a superior labial frenulum tear sustained after minor trauma. Initial examination was reassuring, and he was discharged without laboratory evaluation. On repeat presentation, he was found to have normocytic anemia and isolated prolonged activated partial thromboplastin time. Despite topical and oral antifibrinolytic therapy, he returned with worsening anemia, melena, hematemesis, pallor, and tachycardia. He required a packed red blood cell transfusion, intravenous aminocaproic acid, and hospitalization. Further hematologic evaluation revealed a low factor VIII activity level, confirming mild hemophilia A. There was no prior personal or family history of abnormal bleeding.

**Conclusion:**

This case highlights that hemophilia A may initially present with disproportionate bleeding from a minor mucosal injury, even in the absence of a known bleeding history. Persistent bleeding after minor trauma warrants prompt coagulation evaluation to prevent diagnostic delay and serious complications.

## INTRODUCTION

Labial frenulum tears in infants and toddlers are commonly associated with minor falls and accidental trauma. Such injuries warrant careful evaluation through detailed history-taking and thorough physical examination to exclude nonaccidental trauma. Isolated frenulum tears are typically benign and, like other minor mucosal injuries, often heal rapidly without intervention. However, persistent or excessive bleeding should prompt evaluation for an underlying bleeding disorder.

Hemophilia is an X-linked recessive, inherited coagulopathy that predominantly affects males and is characterized by factor VIII deficiency in hemophilia A and by factor IX deficiency in hemophilia B.^1^ The incidence is approximately one in 5,000 male births, with hemophilia A accounting for about 80% of cases.^2^ Clinical manifestations typically include spontaneous or trauma-induced prolonged bleeding, and an isolated prolonged activated partial thromboplastin time (aPTT) should raise clinical suspicion.^3^ Diagnosis is confirmed by decreased plasma factor VIII or IX activity after exclusion of other coagulation abnormalities, including von Willebrand disease.^4^ Disease severity correlates with factor activity levels and clinical phenotype.^5^ Acute bleeding episodes require urgent treatment with factor replacement therapy.^4^ Although hemophilia is primarily inherited, de novo mutations account for 30–50% of cases.^3^ We present a case of a 14-month-old male patient whose initial manifestation of hemophilia A was a bleeding superior labial frenulum tear, resulting in severe anemia from acute blood loss requiring transfusion and hospitalization.

## CASE REPORT

A previously healthy, fully vaccinated, ex-full term 14-month-old male with a history of prelingual bilateral sensorineural hearing loss presented to the pediatric emergency department (ED) three times within 10 days for persistent bleeding from a superior labial frenulum injury.

His initial visit was for a frenulum injury after biting a plastic baby gate. The patient’s family called the nurse advice line and applied pressure to the area, but when the bleeding continued for approximately five hours despite pressure, the family presented to the pediatric ED. His physical exam on the first ED visit was notable for an upper lip frenulum laceration with minimal bleeding and no indication for repair. There were no other signs of trauma, bruising, or injury. Neurologic, abdominal, and musculoskeletal examinations were unremarkable. Vital signs were stable and appropriate for age. No laboratory testing or imaging was performed, bleeding stopped, and he was discharged with acetaminophen analgesia and anticipatory guidance.

Eight days later he returned to the pediatric ED after his mother found blood and clots on his bedding while he was sleeping. She reported intermittent bleeding from the upper gum since the initial injury but denied new trauma, hematuria, hematochezia, or personal or family history of bleeding disorders. His physical exam was notable for dried blood around the mouth with faint oozing from the upper lip frenulum with a small clot in place. Vital signs remained stable and appropriate for age. Laboratory evaluation revealed a normocytic anemia with hemoglobin 8.4 gm/dL (reference range, 8.0–21.0 gm/dL); hematocrit, 24.2% (33.0–39.0%); mean corpuscular volume (MCV), 80 fL (70–86 fL), prolonged aPTT 42.5 seconds (23.1–38.3 seconds); prothrombin time, 12.9 seconds (12.1–14.5 seconds); international normalized ratio (INR), 0.90 (0.86–1.14 INR); white blood count (WBC), 8.2 ×10^3^/μL (5.0–15.5 ×10^3^/μL); platelet count 327 ×10^3^/μL (250–600 ×10^3^/μL); fibrinogen, 237 (mg/dL) (212–466 mg/dL); D-dimer < 0.27 mcg/mL fibrinogen equivalent units (FEU) (0.09–0.53 mcg/mL FEU); and a normal complete metabolic panel (CMP). See table. Pediatric hematology recommended factor assays (VIII, IX, and XIII) and antifibrinolytic therapy. Topical thrombin was applied to the upper lip frenulum, but the patient spit it out and bleeding continued; he was then given oral aminocaproic acid. Two hours later the bleeding stopped, and the patient was discharged home with a five-day course of oral aminocaproic acid four times daily and pediatrician follow-up.

The patient re-presented to the pediatric ED 26 hours later for the third time with continued bleeding. Despite four doses of oral aminocaproic acid at home he continued to bleed from the frenulum injury. His mother also endorsed two episodes of black stools, one episode of bloody emesis, decreased activity level, and increased skin pallor. Physical exam was notable for pallor, dried blood on the face, and a blood clot at the upper lip frenulum with oozing around the clot. The patient was mildly tachycardic (146 beats per minute) with all other vital signs stable and appropriate for age. Repeat laboratory testing demonstrated worsening normocytic anemia (hemoglobin 5.2 gm/dL, hematocrit 15.1%, MCV 80 fL); normal WBC (8.4 ×10^3^/μL); mild thrombocytopenia platelet count (246 ×10^3^/μL) and a normal CMP. He received 10 mL/kg packed red blood cells over three hours and an intravenous (IV) dose of aminocaproic acid (50 mg/kg). The patient was admitted to the pediatric inpatient service with hematology consultation for severe anemia secondary to acute blood loss. After 24 hours of IV aminocaproic acid and one blood transfusion, his hemoglobin improved to 8.1 gm/dL. The patient returned to functional baseline without further evidence of bleeding. He remained clinically stable with vital signs appropriate for age and was discharged home after 24 hours with a five-day course of aminocaproic acid, outpatient hematology follow-up, and family education.

*CPC-EM Capsule*

What do we already know about this clinical entity?

*Hemophilia A causes prolonged bleeding, but mild disease may remain undiagnosed until minor injury and may mimic nonaccidental trauma.*

What makes this presentation of disease reportable?

*An accidental, isolated labial frenulum tear led to transfusion-requiring anemia and diagnosis of previously unrecognized mild hemophilia A in a healthy toddler.*

What is the major learning point?

*Persistent bleeding after minor trauma should prompt evaluation for an underlying bleeding disorder even with no family history.*

How might this improve emergency medicine practice?

*Early coagulation evaluation in children with persistent mucosal bleeding may prevent diagnostic delays and inappropriate concerm for nonaccidental trauma.*

Further hematologic evaluation demonstrated a low factor VIII activity level (11% [reference range, 55–200%]), confirming the diagnosis of mild hemophilia A. The remainder of his hematology workup was normal with a negative factor VIII inhibitor screen; normal factor IX activity level (91% [55–150%]); and normal von Willebrand factor (vWf) antigen and activity (vWF antigen 87% [55–200%]; vWF activity 82% [55–200%]). Of note, factor IX activity level and von Willebrand panel testing were sent post transfusion.

## DISCUSSION

Severe hemophilia typically presents in the first months of life with spontaneous bleeding, while mild and moderate disease more commonly presents later in childhood and adolescence following trauma. Although the median age at diagnosis is approximately 36 months for mild hemophilia, this diagnosis should also be considered in younger children with bleeding that is prolonged or disproportionate to the reported mechanism of injury, even in the absence of a known family history.^6^,^7^ While most patients with hemophilia A have a prolonged aPTT, a normal aPTT does not exclude mild disease. Measurement of factor VIII and IX levels should be considered in patients with a normal complete blood count and prothrombin time who have unexplained or disproportionate bleeding even when the aPTT is normal.

Our patient had a factor VIII level of 11%, consistent with mild hemophilia A. Despite undergoing circumcision and routine immunizations without significant bleeding complications, he presented with oral bleeding following a relatively minor trauma. This case illustrates the variable clinical presentation of mild hemophilia and highlights that the absence of significant bleeding during previous procedures does not exclude the diagnosis. The severity of hemophilia A and therefore the severity of hemorrhage is directly correlated to the scarcity of factor VIII. Normal factor VIII levels range between 50–150%. Severe hemophilia is defined as factor VIII activity less than 1%, moderate disease as 2–5%, and mild disease as 6–40%. Although spontaneous bleeding is uncommon in mild hemophilia, affected patients remain at risk for excessive bleeding following relatively insignificant trauma.^4^,^8^ Treatment of acute bleeding in hemophilia A consists of prompt recombinant factor VIII replacement and should be initiated immediately, even before completion of patient assessment. Other adjuvant therapies include desmopressin, tranexamic acid, epsilon aminocaproic acid, and management of factor inhibitors.^3^,^4^,^7^

This case also highlights an important diagnostic consideration when evaluating young children with unexplained bleeding. Frequent falls and minor trauma during early ambulation can result in contusions and hemorrhage in children with hemophilia that may mimic nonaccidental trauma. Our patient presented with a labial frenulum tear, an injury that has been associated with several abusive mechanisms such as forceful feeding, stretching of the lip, or direct blows to the mouth.^9^,^10^ However, isolated frenulum tears are not pathognomonic for abuse and have also been reported frequently after accidental falls in ambulatory children.^11^ Comprehensive history-taking, thorough physical examination, and consideration of underlying bleeding disorders are essential when evaluating pediatric patients with injuries concerning for nonaccidental trauma to ensure accurate diagnosis while maintaining appropriate vigilance for child abuse.

## CONCLUSION

Our patient developed severe anemia from acute blood loss requiring transfusion and hospitalization following multiple emergency department visits for a minor mucosal injury. Prior to presentation, he had no history of abnormal bleeding despite a history of circumcision and no known family history of bleeding disorders. This case highlights the potential severity of bleeding disorders in pediatric patients and underscores that hemophilia may present atypically, even in the setting of a reassuring history. Minor trauma accompanied by excessive or persistent bleeding should prompt evaluation for an underlying bleeding disorder, because early recognition and intervention are critical to preventing morbidity and potentially life-threatening complications.

## Figures and Tables

**Table:** All laboratory results for multiple emergency department visits and admission of toddler diagnosed with mild hemophilia A.

Laboratory Test	Second ED Visit (8 days postinjury)	Third ED Visit (9 days postinjury)	Factor Studies (Resulted after discharge)	Reference Range
WBC	8.2 ×10^3^/μL	8.4 ×10^3^/μL	—	5.0–15.5 ×10^3^/μL
Hemoglobin	**8.4 gm/dL**	**5.2 gm/dL**	—	8.0–21 gm/dL
Hematocrit	**24.2%**	**15.1%**	—	33.0–39.0%
MCV	80 fL	80 fL	—	70–86 fL
Platelets	327 ×10^3^/μL	246 ×10^3^/μL	—	250–600 ×10^3^/μL
aPTT	**42.5 seconds**	—	—	23.1–38.3 seconds
PT	12.9 seconds	—	—	12.1–14.5 seconds
INR	0.90	—	—	0.86–1.14
Fibrinogen	237 mg/dL	—	—	212–466 mg/dL
D-Dimer	<0.27 mcg/mL FEU	—	—	0.09–0.53 mcg/mL FEU
CMP	Normal	Normal	—	Normal
Factor VIII activity level	—	—	**11%**	55–200%
Factor VIII inhibitor screen	—	—	Negative	Negative
Factor IX activity level	—	—	91%	55–150%
Von Willebrand Factor Antigen	—	—	87%	55–200%
Von Willebrand Factor Activity	—	—	82%	55–200%

* Bolded values indicate significant values

*ED*, emergency department; *WBC*, white blood cel; μL, microliters; *gm/dL*, gram per deciliter; *MCV*, mean corpuscular volume; fL, femtoliters; *aPTT*, activated partial thrombin time; *PT*, prothrombin time; *INR*, international normalized ratio; *CMP*, comprehensive metabolic panel; *FEU*, fibrinogen equivalent units.

## References

[1] Soucie JM, Miller CH, Dupervil B (2020). Occurrence rates of haemophilia among males in the United States based on surveillance conducted in specialized haemophilia treatment centres. Haemophilia.

[2] Ferreira N, Proença E, Godinho C (2015). Neonatal hemophilia: a rare presentation. Pediatr Rep.

[3] Chalmers E, Williams M, Brennand J (2011). Paediatric Working Party of the United Kingdom Haemophilia Doctors’ Organisation. Guideline on the management of haemophilia in the fetus and neonate. Br J Haematol.

[4] Salen P, Babiker HM, Hemophilia A StatPearls [Internet].

[5] Franchini M, Mannucci PM (2013). Hemophilia A in the third millennium. Blood Rev.

[6] Kulkarni R, Soucie JM, Lusher J, Haemophilia Treatment Center Network Investigators (2009). Sites of initial bleeding episodes, mode of delivery and age of diagnosis in babies with haemophilia diagnosed before the age of 2 years: a report from the Centers for Disease Control and Prevention’s Universal Data Collection project. Haemophilia.

[7] Centers for Disease Control and Prevention Data and statistics on hemophilia.

[8] Kulkarni R, Soucie JM (2011). Pediatric hemophilia: a review. Semin Thromb Hemost.

[9] Maguire S, Hunter B, Hunter L (2007). Diagnosing abuse: a systematic review of torn frenum and other intra-oral injuries. Arch Dis Child.

[10] Thackeray JD (2007). Frena tears and abusive head injury: a cautionary tale. Pediatr Emerg Care.

[11] Yee R, Kwek VYX, Chng CK (2023). Frenal tears: accidental or non-accidental?. Singapore Med J.

[12] Monagle P, Barnes C, Ignjatovic V (2006). Developmental haemostasis: impact for clinical haemostasis laboratories. Thromb Haemost.

